# BioModelos: A collaborative online system to map species distributions

**DOI:** 10.1371/journal.pone.0214522

**Published:** 2019-03-27

**Authors:** Jorge Velásquez-Tibatá, María H. Olaya-Rodríguez, Daniel López-Lozano, César Gutiérrez, Iván González, María C. Londoño-Murcia

**Affiliations:** Laboratory of Applied Biogeography, The Alexander von Humboldt Institute for Research on Biological Resources, Bogotá D.C., Colombia; Aristotle University of Thessaloniki, GREECE

## Abstract

Information on species distribution is recognized as a crucial input for biodiversity conservation and management. To that end, considerable resources have been dedicated towards increasing the quantity and availability of species occurrence data, boosting their use in species distribution modeling and online platforms for their dissemination. Currently, those platforms face the challenge of bringing biology into modeling by making informed decisions that result in meaningful models, based on limited occurrence and ecological data. Here we describe BioModelos, a modeling approach supported by an online system and a core team of modelers, whereby a network of experts contributes to the development of species distribution models by assessing the quality of occurrence data, identifying potentially limiting environmental variables, establishing species’ accessible areas and validating modeling predictions qualitatively. Models developed through BioModelos become freely and publicly available once validated by experts, furthering their use in conservation applications. Our approach has been implemented in Colombia since 2013 and it currently consist of a network of nearly 500 experts that collaboratively contribute to enhance the knowledge on the distribution of a growing number of species and it has aided the development of several decision support products such as national risk assessments and biodiversity compensation manuals. BioModelos is an example of operationalization of an essential biodiversity variable at a national level through the implementation of a research infrastructure that enhances the value of open access species data.

## Introduction

Species distributions are an essential biodiversity variable (EBV) [1, 2], critical to evaluate species’ conservation status and trends [3, 4], measure biodiversity change [5–7], guide conservation and management at the species and community levels [8] as well as to assess their ecosystem services [9], potential impacts on human activities [10, 11] and health [12, 13]. This EBV is also a key input for the calculation of indicators to evaluate countries' progress towards achieving international targets, such as the Convention on Biological Diversity Aichi targets [14] and the United Nations’ Sustainable Development Goals (SDGs) [15]. For example, according to the Biodiversity Indicators Partnership (www.bipindicators.net), 11 out of 20 Aichi targets and 6 out of 17 SDGs use indicators that require information on species distribution, either for their calculation or for their disaggregation at national and subnational levels. Thus platforms that consolidate and facilitate access to the highest quality data on species distributions are necessary to coordinate biodiversity observation delivery as EBVs and aid biodiversity conservation and management globally [16].

Considerable international efforts and resources have been designated towards the mobilization of primary biodiversity data (PBD), particularly through the Global Biodiversity Information Facility (GBIF). These data are fundamental for many conservation analyses based on species distributions. However, for most areas in the world, our knowledge on species distributions based on PBD is geographically biased and incomplete [17]. This situation is particularly dire in biodiversity hotspots which lack sufficient information on species distributions based on PBD even at coarse spatial scales [17–19]. Therefore, for most regions on earth, methods that generalize occurrences to areas representing species distributions are necessary to use PBD in conservation applications.

Species distribution modeling has emerged in the last two decades as a set of methods and practices to estimate species distributions [20–22]. They are based on PBD and environmental data and use a variety of statistical methods to infer the probability of occurrence or suitability in unsampled sites. As such, they are a powerful tool to overcome the Wallacean shortfall (i.e. the lack of knowledge on species geographic distributions [23]), due to their ability to produce reasonable predictions with few occurrences [24, 25], repeatability and ease of update. However, their implementation is not straightforward [26–28] and fully automated, large scale modeling procedures face several challenges [29, 30], namely the need of expert’s knowledge to detect and correct certain types of errors in PBD [31]; select meaningful environmental covariates [32, 33]; determine each species’ accessible area [34, 35] and judge the biological realism of predictions [36].

Current platforms that provide maps of species' distributions are either based largely on expert maps (e.g. map of life, www.mol.org) or use fully automated modeling workflows (e.g. BIEN, http://biendata.org/). Expert maps in some cases may be the only way to characterize a species’ distribution, for example when there are very few observations available, but are difficult to update as new observations accumulate [37], they are not repeatable and their precision is often too coarse to inform conservation at regional scales [38]. On the other hand, large scale, fully automated modeling workflows are unable to detect and fix errors that require domain specific expertise (e.g. species misidentification or geographic outlier detection [31]) and unspecific modeling choices, for example of accessible areas and environmental variables, is likely to result in biologically unrealistic models.

To address the challenge of mapping large numbers of species without compromising biological realism we devised BioModelos (biomodelos.humboldt.org.co), an online system that involves a network of experts and a core team of modelers in the development and validation of species distribution models, which are freely available for public visualization and download. Here we describe the operational approach of BioModelos, the functionalities of its web app and its implementation in Colombia. Although BioModelos has thus far been deployed in a single country, our philosophy and software architecture can may be applied to other regions and even scaled up to global implementations.

## Network structure and governance

The aim of BioModelos is to provide distribution maps for a set of species in a particular area that are validated in terms of their biological realism by experts. To that end, in BioModelos experts are arranged into groups according to their areas of taxonomic and/or geographic expertise. Experts are defined as individuals whom are able to either curate and improve the taxonomic and/or geographic quality of occurrence data, inform the selection of certain modeling parameters (e.g. accessible area) or assess the performance of competing species distribution hypothesis, for at least one species in their group.

Each group is coordinated by one or more moderators, whom are ideally well-connected members of a community of researchers interested in advancing the knowledge on the distribution of a set of species. As such, they are responsible for the objective evaluation of the expertise of potential members, setting deadlines for each step in the model development workflow in agreement with group experts and expedite the completion of the group modeling agenda.

The core team of BioModelos, facilitates some of the steps in the modeling workflow according to the group needs, namely aggregating species occurrences and running automated data validation routines, modeling species distributions and processing expert’s feedback on models. Additionally, the core team approves the creation of new groups, enables the publication of models generated by third parties and updates occurrence databases following recommendations provided by the groups.

## Model development workflow

**Fig 1:**
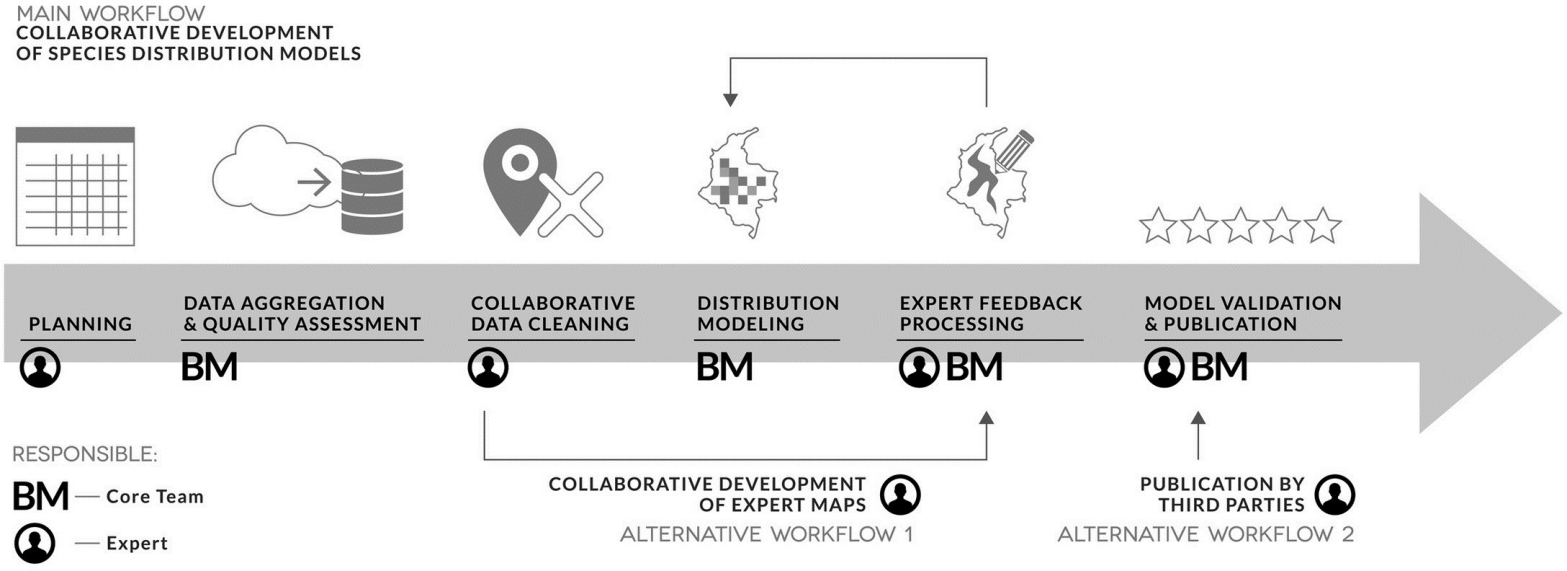
Species modeling workflow.

Species distribution hypothesis available in the BioModelos web app are generated either by collaborative development of species distribution models and expert maps or by third parties that independently upload models to the web app (Fig 1).

### Collaborative development of species distribution models

#### Planning

In BioModelos, every species is associated to a single group of experts whom are tasked with generating, improving or validating a distribution hypothesis for each species in the group. Experts within a given group inform the core team which species they plan to model, propose deadlines for modeling activities and provide information that may be relevant for model development, such as previously curated occurrence data or private data, suggestions of meaningful environmental variables to consider in modeling, among others.

#### Data aggregation and quality assessment

Unless the group provides previously curated data, the core team aggregates occurrences from multiple data providers (GBIF, eBird, VertNet, speciesLink etc.), either manually or through web services when available. After aggregation and standardization, a series of automated data quality checks are performed (S1 Table). Importantly, a permanent unique identifier for each occurrence is generated and original identifiers (e.g. occurrence id, institution, collection code, catalog numbers etc.) are maintained throughout the process so that their provenance may be traced and feedback on data quality may be sent back to data providers whenever mechanisms to that end exist. Finally, occurrences and quality assessment information is added to the BioModelos’ contents database.

#### Collaborative data cleaning

Records that pass selected filters based on the automated quality checks become visible on the BioModelos geographic viewer. When there are spatial duplicates (i.e. more than one record falls in a 1 km cell), only the most documented record is visible. The filters implemented for each occurrence dataset are variable, depending on its characteristics and the amount of records available for a particular group (Table 1). Published records are further reviewed in BioModelos by experts to identify and flag likely identification and georeferencing errors not detectable in automated checks. Whenever corrections are possible, experts are encouraged to edit identifications or coordinates. If a correction is not possible, experts flag records according to the manual flags presented in S1 Table. All changes in the contents database are automatically logged so that is possible to track and revert changes made to any particular record.

**Table 1:** Summary of BioModelos expert network structure and model development status.

Group name	Number of experts	Species in group	Model development status (spp.)		
			Under development	Pending validation	Validated
Bees of Colombia	12	16			
Aquatic birds of Colombia	48	80	2		14
Birds of Colombia	36	277	49	156	4
Beetles	18	4	1		
Introduced fauna	24	21	17		1
Plants of paramo	21	212	212		
Herps of Colombia	60	67	60	1	2
Introduced plants of Colombia	33	32	31		
Dragonflies Colombia	5	13	11		
Magnolias of Colombia	17	34	17	13	
Mammals of Colombia	81	52	6	1	
Orchids of Colombia	25	5			
Palms	8	51	46		
Freshwater fishes	10	1	1		
Carnivorous plants of Colombia	4	4	1		
Plants of dry forest	28	53	48		
Primates of Colombia	28	38		1	37
Zamias of Colombia	17	20			20
**TOTAL**	**475**	**980**	**502**	**172**	**78**

To inform the development of distribution models, experts are also asked at this step to delineate rough polygons of species’ accessible areas (M *sensu* [39]) using a polygon tool and identify land cover types where species are expected to maintain viable populations by filling out a habitat preferences form.

#### Distribution modeling

After occurrence data has been assessed for quality, the BioModelos core team develops distribution models using occurrences without quality issues. Our modeling workflow consists broadly of the following steps: (1) occurrence thinning [40], for which a threshold is selected based on exploratory analysis of data availability and spatial autocorrelation; (2) environmental data selection depending on species’ biology and dataset quality; (3) selection of modeling method based on available occurrences (1–2: 10 km buffer around points; 3–5: convex hull; 5–9: Bioclim; >9: MaxEnt); (4) sampling background data from accessible areas, from target groups or bias sampling surfaces whenever sufficient sampling exists [41], otherwise at random; (5) use spatial partitioning to evaluate and optimize MaxEnt model parameters through the ENMEval package [28] and evaluate Bioclim models; (6) develop distribution models using the full set of occurrences and for MaxEnt the regularization and feature settings that optimize performance; (7) generation of thresholded models at the minimum, 10th, 20th and 30th percentile training presence; (8) upload metadata for each model to the contents database and model predictions as GeoTIFF files to the BioModelos front-end where they become visible at this stage under status “under development”.

Although many alternative workflows and modeling choices could be made [27], this workflow adequates well to the availability of data in our country of implementation and similar workflows have been implemented elsewhere in the SDM literature (e.g. [42]). Nonetheless, we emphasize that the BioModelos web app acts as a layer through which information from experts is gathered, but once collected, a variety of modeling workflows may be implemented. This allows the core team to update its modeling workflow based on advances in the field independently from the BioModelos web app.

#### Expert feedback processing

**Fig 2:**
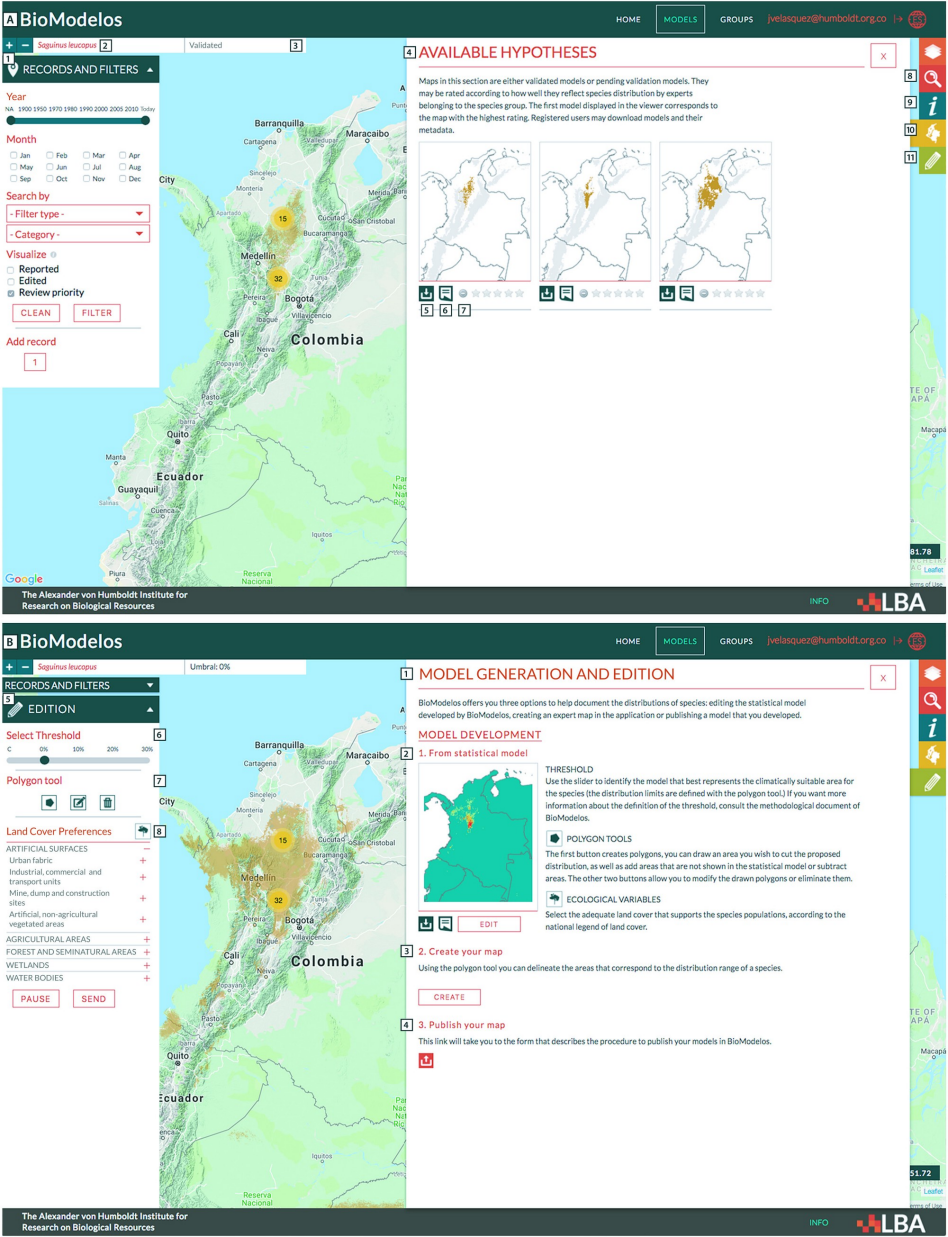
A. BioModelos geographic viewer subcomponents. (1) Occurrence filter panel; (2) Species name; (3) Model status; (4) Available hypothesis box; (5) Download link; (6) Metadata link; (7) Score model; (8) Advanced search box; (9) Species fact-sheet box; (10) Available hypothesis box; (11) Map generation and editing box. B. BioModelos model generation and input tools (only visible to species’ experts). (1) Model generation box; (2) Model editing option; (3) Create map option; (4) Publish map option; (5) Model editing panel; (6) Threshold slider; (7) polygon tool to delimit accessible areas and identification of areas of model over and underprediction; (8) species habitat preferences form.

Models published in the previous step are reviewed by experts whom select through a slider a omission threshold (varying from 0% to 30%) to convert continuous models into binary models. Though other thresholding methods could be easily implemented in the web app [43, 44], our current range of thresholds has a straightforward interpretation by experts and is sufficient to elicit their opinion on species’ prevalence within an area of interest based on presence-only data. In addition, experts may further refine thresholded models using a polygon tool to delineate areas of model over or under-prediction. Using thie feedback, models are edited offline by the BioModelos core team and the resulting models are published in the “available hypothesis” box of BioModelos under status “pending validation” (Fig 2).

### Collaborative development of expert maps

For many species without sufficient occurrence data it is not possible to develop a distribution model. In those cases, experts may still use BioModelos to inform the general range limits of a species and their habitat preferences using the “create your map” feature (Fig 2). By choosing this option, experts can also provide instructions to further refine them based on geographic features such as barriers (rivers, canyons, watersheds) and elevation. These instructions are processed by the core team to generate species distribution maps that are displayed in the BioModelos viewer in the “available hypothesis” box.

### Publication by third parties

As a rapidly expanding field, a large number of species distribution models are being generated by numerous researchers. Many of these are produced by experts on the species being modeled, and thus represent a valuable resource to other scientists and users. The publication of these models is facilitated in BioModelos through a publication option in the model generation box (Fig 2B) that allows users to submit their models in raster format, along with occurrence data (optional), model metadata and methods (templates in spanish available at http://biomodelos.humboldt.org.co/guia_documentacion_y_plantillas.zip). Whenever a submission consists of models used in previously peer-reviewed research, models are published in BioModelos without further review. Otherwise the methodology is reviewed by the core team or an external reviewer and a decision is made regarding its publication.

## Model validation and publication

An essential feature of BioModelos is the validation of species’ distribution hypotheses by experts. This validation is inherently qualitative and it is made on the basis of experts’ subjective judgement on the biological realism of a model. Since at any point there may be several hypotheses for the distribution of a species (for example a species may have a published model and a collaborative model), we ask experts in each group to score the models for species in their group, on a qualitative scale from 1 to 5 (1: no credibility, 5: complete credibility). Models with an average score of 3 or higher are approved and flagged as “*validated*”. If no model is approved, experts may either decide to go back and modify their inputs or suggest the development of a new model altogether. The process of expert model evaluation is repeated whenever new distribution hypotheses are generated, taxonomy changes or significantly new occurrence data becomes available. Once a species has a validated model within BioModelos, the core team calculates a number of statistics based on its distribution to aid the assessment of its conservation status and trends. These statistics are displayed in the species fact-sheet box (Fig 2).

All distribution models visible in BioModelos are available for download in GeoTIFF format at their original resolution and their use and distribution is regulated through Creative Commons 3.0 licenses. Everyone involved in data cleaning, generation of model inputs and validation of models is recognized as a model author. Besides including all author information, our metadata standard for models and occurrences, contains all relevant information pertaining to data sources, model development (including links to modeling logs in GitHub) and performance statistics.

## Web application architecture and components

**Fig 3:**
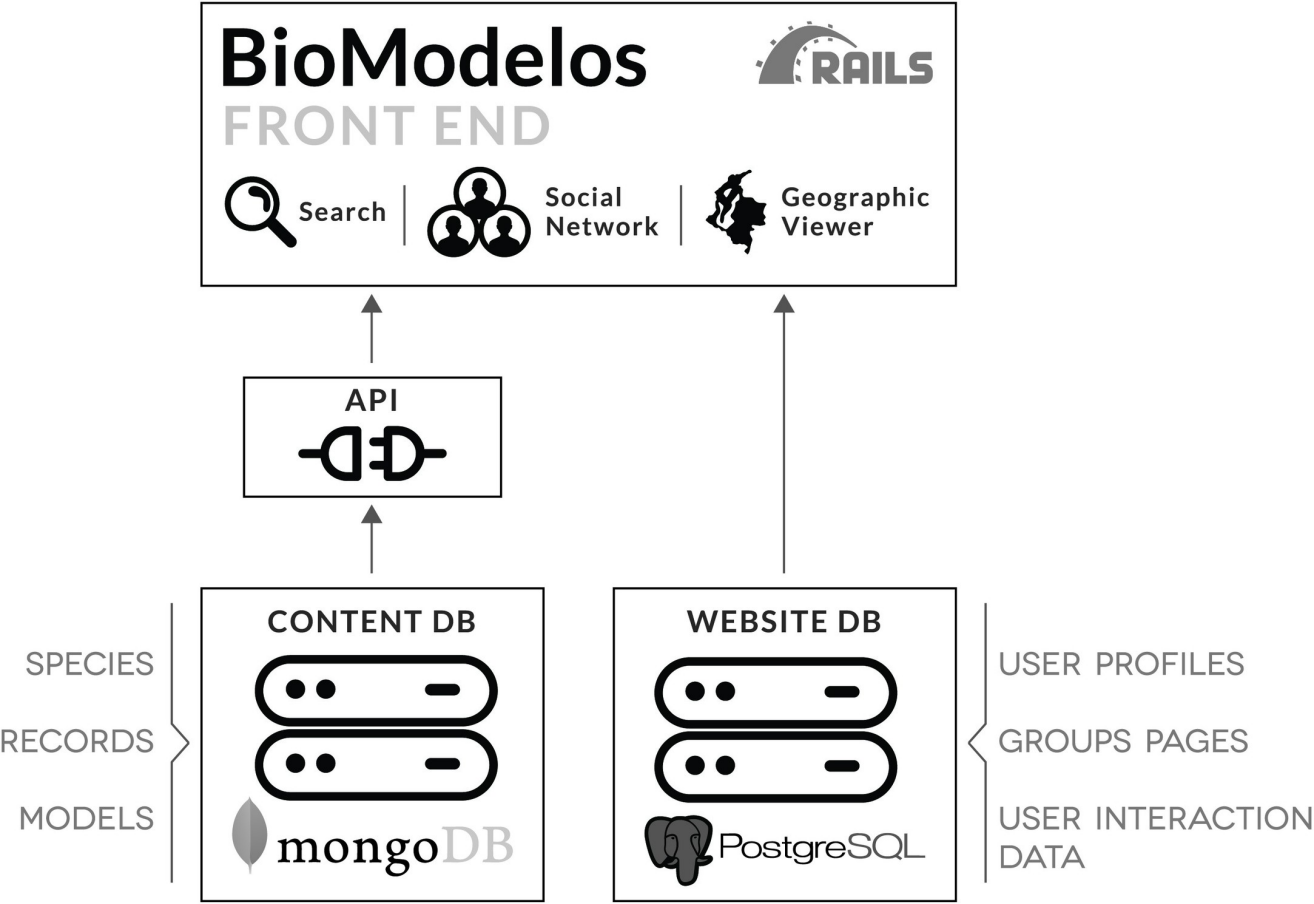
BioModelos web application components and architecture.

BioModelos has been developed as an open source web application composed of four main components over a three-tier layered architecture: two independent databases (contents and website), an API (Application Programming Interface) and the web application front-end (Fig 3). The contents database was developed following a non-relational scheme in MongoDB and it includes the collections “species” (keeps the taxonomic backbone and species’ ancillary information), “records” (occurrence data and quality assessment information) and “models” (model metadata and species’ distribution derived statistics). The website database was developed using a relational scheme via PostgreSQL and it stores all relevant information about the interaction of the users within the website, such as users, groups, user created layers, ratings, tasks, publications, and downloads among others. The website database is directly connected to the BioModelos front-end while the content database is accessible through the web services implemented in the API. This architecture allows BioModelos to scale, grow and distribute each database independently as well as to store user interactions on the website privately while opening the BioModelos content database to third-party applications using the API.

The front-end of BioModelos was developed using the Ruby on Rails framework including Javascript libraries supporting some of the functionalities. It also stores all the static files (e.g. images, map files, documents) needed to display the distribution models. The front-end consist of three main components: a search engine, a social network and a geographic viewer. The search engine allows users to find either species distributions by entering their scientific name or sets of species based on attributes using the advanced search functions. The social network component comprises expert’s public profiles and group profiles and allows interaction among experts using built in messaging tools. It also facilitates the monitoring of progress in completing particular modeling tasks using the task dashboard, and approval for the admission of new group members by group moderators. Lastly, most user interactions take place in the geographic viewer. It contains tools related to clean data, provide feedback on distribution models and visualize distribution hypotheses, model statistics and metadata. A complete description of these functionalities is available in S2 Table.

## Implementation of BioModelos in Colombia

The BioModelos web app and network have both been under simultaneous development since 2013. During this time, we have conducted 15 workshops with experts that have been essential to consolidate expert groups as well as to gather feedback to enhance user experience in the web app and improve the modeling workflow. Currently, out of 1052 registered users, there are 475 experts associated to 20 expert groups, which in turn are managed by 34 moderators (Table 1; data as from October 2018). Collectively, these experts are tasked with contributing to the development of 980 SDMs. Additionally, 17 expert maps and 216 models have been published since the model publishing feature was implemented in January 2017.

User registration to download models has been mandatory from August 2017. Ever since, 430 downloads have been made in BioModelos, 33% of them for academic research, 32% for educational activities and the remaining for applied research, environmental consulting, bioprospecting and other activities. Validated models have allowed Humboldt Institute (BioModelos’ host institution in Colombia) to support conservation decision making by informing plans to compensate biodiversity loss [45], elaborate species’ extinction risk assessments [46] and the generation of official cartography for Colombia through the biotic component of the Colombian ecosystems map [47].

## Discussion

We presented BioModelos, an approach to facilitate collaboration between experts (e.g. field biologists, ecologists, taxonomists, biogeographers and modelers) to generate publicly available information on species distribution mediated by a core team and a web app. By involving experts in the development of models, we aim to fill the gaps in primary biodiversity data and assess the biological realism of model predictions by eliciting experts’ opinion on species distribution as well as to avoid the prevalent duplication of efforts in data cleaning and modeling [48]. Both of these features are necessary to advance faster towards the amelioration of the Wallacean shortfall as well as to further the use of SDMs to generate EBV that inform conservation decision making processes [15].

Species distribution modeling is still a very dynamic field in which novel methods and recommendations arise frequently (e.g. [27]). By keeping the expert-opinion data gathering process independent from the modeling process, we have been able to implement multiple modeling workflows with little impact on the design of the BioModelos user interface or experience, keeping the app maintenance costs low. This feature will allow us to continue to refine our modeling workflow in the future, for example by using methods that formally incorporate expert opinion in model development [49] and integrating established semi-automated modeling workflows, such as Wallace [42].

The difficulty of evaluating the accuracy of species distribution models in presence-only models has long been recognized and discussed [50, 51]. Simply put, traditional performance metrics (e.g. AUC, TSS) of models built based on presence-only data may only tell us how well a model prediction discriminates presences from arbitrary pseudo-absences and their value and statistical significance depends on how those pseudo-absences are drawn [52, 53]. Therefore, these metrics are a measure of relative performance [34] not suitable for comparison among species and of difficult interpretation for interested users of model predictions without a modeling background. An important feature of BioModelos is that besides providing standard measures of model performance for each published model it also encourages the subjective evaluation of models by experts. This qualitative evaluation together with authorship information on the experts that validate the model may help end-users to decide whether to use a model or not in a particular application [54].

An important challenge of the implementation of BioModelos in Colombia has been to motivate the autonomous completion of modeling agendas by experts’ groups. Thus far, the most successful collaborative modeling experiences have been in groups that require BioModelos outputs (i.e. models and species fact-sheets) for species risk assessments [46] or action plans (Cycads [55]; Magnoliaceae and Primates, *in prep*). Also, the publication of models developed by third-parties has contributed an important proportion of the models available in BioModelos and we continue to encourage model publication by contacting modelers identified through publications and scientific events. However, for the remaining species we are still in the process of devising incentives to increase the participation of experts in BioModelos, such as the development of electronic publications that are formally recognized as research products in academic performance reviews.

The BioModelos web app is open source (https://github.com/LBAB-Humboldt/BioModelos.v2) and its use by any interested party is permitted through a MIT License. However, due to the technical requirements for its installation and maintenance, the collaborative nature of the BioModelos approach and the need of a core team consisting at least of a modeler and a network manager for its implementation, it is best suited for national level implementations hosted by research institutions. Although this geographical scale may seem arbitrary as species are not limited *per se* by national boundaries, it is practical considering that many uses of models for conservation decision making take place at national and subnational levels and that experts usually confine their expertise to countries due to accessibility, funding restrictions and ease to obtain research permits. Hence, by implementing BioModelos at a national scale, we contribute both to increase occurrence data fitness for use in distribution modeling, potentially aiding global modeling initiatives once mechanisms to collect error reports through web services are implemented in data providers such as GBIF [48] and to the consolidation of a global coordinated monitoring system of species distributions through National Biodiversity Observation Networks [56], to make EBVs genuinely global [57].

## Supporting information

S1 Table Automated and expert data validation and cleaning procedures in BioModelos. (DOCX)

S2 Table BioModelos’ main features. (DOCX)
