# Supplementary material for: BioModelos: A collaborative online system to map species distributions
Source: PLoS One. 2019 Mar 27;14(3):e0214522. doi: 10.1371/journal.pone.0214522 (PMC6436796; doi:10.1371/journal.pone.0214522)
Supplement: S1 Table — (DOCX) [file pone.0214522.s001.docx]

**Table S2.** BioModelos main features

| **Feature** | **Description** |
| --- | --- |
| Occurrence data visualization | |
| Filter by occurrence data attributes | Occurrence data may be filtered by date (month, year), basis of record, data source and institution. |
| Filter by quality tags | Occurrence data may be filtered to show only those that have been flagged for quality issues. |
| Occurrence data management and quality assessment | |
| Add record | Allows the addition of one record to the occurrence collection Required fields are species (populated automatically), latitude & longitude (entered manually or through the viewer) and basis of record. |
| Update record | Allows the correction of geographic fields (latitude, longitude and locality) as well as the taxonomy (scientific name) of a record in the occurrence collection. |
| Report record | Report a geographic or taxonomic accuracy issue with an existing record. Considered flags are listed in Table 1. |
| Distribution hypothesis generation | |
| Edit model | This set tool allows experts to convert a continuous model into a binary one and edit it to better reflect a species current distribution based on climate and vegetation. Included functions allow users to:   - Select an omission threshold to develop binary model. - Draw, edit or erase a polygon to identify accessible areas and areas of model over or under prediction. - Specify the altitudinal limits of the species. - Select species’ habitat preferences (based on the corine land cover - level 3 legend). |
| Distribution hypothesis validation | |
| Score | Allows users to assess how well a distribution hypothesis represents its current distribution. The scores provided by the species experts’ are used to establish whether a species distribution hypothesis is approved or not. |
| Species statistics and indicators | |
| Statistics/indicator viewer (tab, module?) | Displays statistics/indicators on approved models. Currently supports:   - Range size (original and current) - Extent of occurrence (based on models or occurrences) - Representation of species distribution in three protected area types. - % Distribution in indigenous lands. - % Land cover types within distribution (filter by suitable land covers). - Recent and projected trends in forest cover within distribution. |
| Group page | |
| Statistics | Group descriptive and performance statistics. Expert contribution ranking. |
| Species/Experts tab | Lists the species and experts that are associated with the group. |
| Tasks | Dashboard to assign (group owner) and mark as completed specific tasks (review occurrences, edit model, select suitable land cover, approve model) to an expert-species pair. |
| Networking | Set of features that facilitate the interaction among group members:   - Send message to group owner. - Send message to all group members (owner only). - Join group/Abandon group. |
| Expert Profile page | |
| Statistics | Expert descriptive and performance statistics. |
| Species | Lists of species associated with user |
| Tasks | Dashboard to record progress towards achieving assigned tasks for each species. |
| My maps | Edits to models done by expert (private) and consensus models in which the expert has participated (public) |
| Activity | Timeline of user activity on site. Allows user to delete specific contributions. |
